# The Safety and Efficacy of Repetitive Transcranial Magnetic Stimulation in the Treatment of Major Depression Among Children and Adolescents: A Systematic Review

**DOI:** 10.7759/cureus.14564

**Published:** 2021-04-19

**Authors:** Pradipta Majumder, Sabish Balan, Vikas Gupta, Roopma Wadhwa, Tarique D Perera

**Affiliations:** 1 Psychiatry, WellSpan, York, USA; 2 Psychiatry, Harlem Hospital, New York, USA; 3 Psychiatry, South Carolina Department of Mental Health, Columbia, USA; 4 Psychiatry and Behavioral Sciences, South Carolina Department of Mental Health, South Carolina, USA; 5 Psychiatry and Behavioral Sciences, Contemporary Care, Greenwich, USA

**Keywords:** transcranial magnetic stimulation, adolescent, pediatric, depression, randomized control trial

## Abstract

Major depression is a chronic debilitating condition affecting people of all ages and is rising over the past decade. Major depression among children and adolescents is often resistant to traditional treatments, thus necessitating the exploration of novel strategies. Repetitive transcranial magnetic stimulation (rTMS) is gaining increasing attention as a useful tool in treating various conditions and has received the US Food and Drug Administration (FDA) approval to treat depression and obsessive-compulsive disorder among adults. Favorable outcomes among adults generated interest in using it among children. Until recently, the existing literature lacked randomized sham-controlled trials on this topic among children and adolescents. The newest additions in the literature necessitated another in-depth look at the data to explore the safety and efficacy of rTMS in the context of depression among children and adolescents. We searched the Medline and Cochrane databases and included 18 articles for our systematic review. Our systematic review indicates level 1 evidence that rTMS is safe but failed to show its superiority to placebo as a stand-alone treatment for resistant depression among children and adolescents. However, there is level 2 evidence favoring add-on rTMS to treat major depression among children and adolescents. The study subjects appear to tolerate the rTMS treatment well with some minor and mostly self-limited side effects. Risks of treatment-emergent hypomanic symptoms and seizure appear to be very low. There is no evidence of worsening of suicidal ideation or cognitive decline during rTMS treatment.

## Introduction and background

The rate of major depression has increased from 8.7% in 2005 to 11.3% in 2014 in adolescents [1]. Major depression in childhood is often associated with school dropout, unemployment, and unwanted pregnancy [2]. The Treatment for Adolescents with Depression Study showed that a combination of fluoxetine and cognitive-behavioral therapy was associated with a higher remission rate in adolescent depression than either treatment alone [3]. Nevertheless, almost 30-40% of depressed adolescents fail to respond to traditional treatment [4,5]. There are also concerns about treatment-emergent side effects, such as suicidality with the use of antidepressants [6,7]. The practice parameter of the American Association of Child and Adolescent Psychiatry recommends electroconvulsive therapy (ECT) for adolescents in resistant cases of major depression [8]. However, ECT is less frequently used [4,9] due to cognitive side effects, prolonged seizure, and anesthetic complications [4,10]. As a result, there is an increasing interest in novel treatment approaches for major depression.

Repetitive transcranial magnetic stimulation (rTMS) is a relatively new treatment modality that is gaining attention in the treatment of various conditions afflicting adults [11-13]. Three large, multi-site, randomized, sham-controlled trials amongst medication-free depressed adults who failed to respond to antidepressant trials have demonstrated its efficacy in major depression [14-16]. A recent systematic review indicates a better response rate among adults with fewer previous failed antidepressant trials [17]. The response rate can be as high as 95.5% and a remission rate of 68.2% in the first episode [18].

The US FDA approved the use of rTMS for major depression and obsessive-compulsive disorder among adults who failed to respond to medications [13,19]. Adult literature indicates that the most common side-effects of rTMS are scalp tenderness and headache [11,20]. Serious side-effects such as seizure and treatment-emergent mania or hypomania are rare [20,21]. The adult literature generated a growing interest in investigating its role in depression among children and adolescents. The current systematic review explores the safety and efficacy of rTMS in the case of major depression among children and adolescents.

## Review

Methods

**Figure 1:**
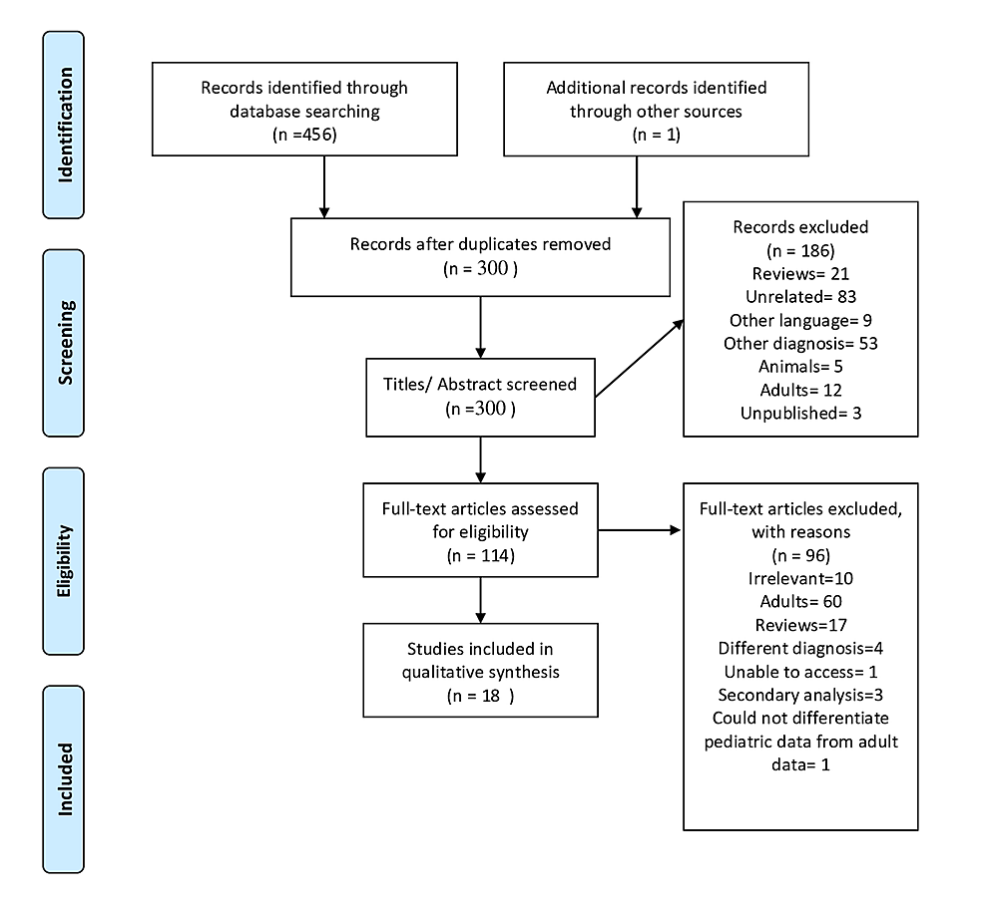
PRISMA 2009 Flow Diagram PRISMA: Preferred Reporting Items for Systematic Reviews and Meta-Analyses. The image was obtained from Moher et al. [23].

The systematic review aimed to collate various open-label studies, randomized controlled trials (RCTs), case reports, case series investigating the safety and efficacy of rTMS in treating major depression among children and adolescents (age≤18). Studies that included children and adults but focused on adults or did not present data of children and adolescents separately were not included. We searched the Medline and Cochrane databases and kept various MeSH keywords such as “pediatric,” “adolescent,” “depression,” “rTMS” in varying combinations. Additionally, we searched the reference lists of various articles and reviews using the same search engine and contacted other researchers to gather additional information about their published papers. We included literature in the English language published until November 2020. We excluded studies whose primary diagnosis was not major depression and those who used other variety of TMS. We have utilized the Joanna Briggs Institute quality appraisal tool to assess the quality of the included studies [22]**. **One of the authors (PM), in consultation with two other authors (SB and TP), selected the articles for the systematic review and followed the PRISMA guideline (Figure 1). We did not attempt a quantitative synthesis with the data due to the heterogeneity of studies and outcome parameters/assessment methods.

Results

Using the various combinations of keywords, we retrieved 457 articles. Removal of duplicates resulted in 300 publications. All these 300 articles were screened based upon the titles and abstracts. We excluded 186 articles due to various reasons (as shown in Figure 1). We retrieved 114 full texts for eligibility amongst which, 96 were excluded based on relevance (N 10), involving adults (N 60), reviews (N 17), different diagnosis (N 4), unable to access (N 1), secondary analysis (N 3), and could not differentiate pediatric data from adult data (N 1). Finally, 18 articles, including 13 multi-subject trials and 5 case reports, were incorporated in this systematic review. We identified another three studies [24-26] that incorporated data from previous studies already included in this systematic review. We have discussed their findings.

Characteristics of the Studies

Tables 1 and 2 summarized the characteristics of the studies. The studies include open-label trials [27-32], case reports [33-37], case series [38-41], naturalistic studies [42], and randomized sham-controlled trial [43]. One study used emailed questionnaires to a Worldwide pool of rTMS centers [44]. The majority of the studies are from the USA [28-31,33-35,40,43]. The rest of the studies were from China [37,38,42], Israel [32], Finland [36], Canada [27,39], Australia [41], and across multiple nations [44]. The lowest age of the recipient of rTMS was ten [40,42,43], but most of the patients were above 12.

**Table 1:** Study design, intervention, and efficacy multi-subject trials MINI: Mini International Neuropsychiatric Interview, MINI-KID: Mini International Neuropsychiatric Interview for Children, YMRS: Young Mania Rating Scale, K-SADS-PL: Kiddie Schedule for Affective Disorders and Schizophrenia Present and Lifetime version, CDRS-R: Children’s Depression Rating Scale Revised, HAM-D: Hamilton Depression Rating Scale, HAM-A: Hamilton Anxiety Rating Scale, BDI: Beck Depression Inventory, C-SSRS: Columbia Suicide Severity Rating Scale, CDRS-R: Depression Rating Scale, Revised, BSI-CV: Beck Scale for Suicide Ideation-Chinese Version, MADRS: Montgomery–Asberg Depression Rating Scale, QIDS-A17-SR: Quick Inventory for Depressive Symptomatology Adolescent Seventeen Item Self Report, CGI-S: Clinical Global Impressions-Severity and CGI-I: Clinical Global Impressions-Improvement scales, ATHF: Antidepressant Treatment History Form, CAVLT-2: Children’s Auditory Verbal Learning Test-2, D-KEFS: Delis–Kaplan Executive Function System, SIQ: Suicidal Ideation Questionnaire, SCARED: Screen for Child Anxiety-Related Disorders questionnaire, CNTA: Cambridge Neuropsychological Test Automated Battery, CES-DC: Centre for Epidemiological Studies–Depression Scale for Child, RAVLT: Rey Auditory Verbal Learning Test, TMT: Trail Making Test A and B, COWAT: Controlled Oral Word Association Test – letter and category, BQ: Beck’s Questionnaire. *The authors presented three cases in the original article. However, one out of the three cases was a healthy control. The healthy control was excluded from this systematic review. Pediatric TMS Safety and Tolerability Measure.

Author	Study design	Rating scales	N (age)	Number of sessions	Session duration (Sec per train, sec per duration)	Intensity %MT	Reported side effects	Efficacy/effectiveness
Croarkin et al. [43]	Randomized, sham-controlled	HAM-D, MADRS, CDRS-R, CGI-S, QIDS-A-17-SR, CSSR-S, YMRS	103 (12–22) rTMS group-48 sham-controlled group-55	30	75 trains, 10 pulses per second over 4 seconds, intertrain interval of 26 seconds, 10 Hz, 3000 pulses per session	120	Suicidal ideation, classified as not related to rTMS headache, nausea, facial twitching	Response rate is 41.7% (remission rate 29.2%) in the active treatment group and 36.4% (remission rate 29%) in sham-controlled group
Zhang et al. [42]	Naturalistic study	HAM-D, HAM-A	42 adolescents vs. 75 adults (10–80)	11–20	80 trains, 30 pulses per train, 10Hz, 12 seconds intertrain-interval, 2400 pulses per session	120	Transient headache, musculoskeletal discomfort	All age groups showed improvement in both anxiety and depression. Symptom improvement more among adolescents. Significant time-effect was seen 94.1% of adolescents responded, and 88.2% achieved remission at the end of 4 weeks
MacMaster et al. [27]	Open level	K-SADS-PL, CDRS-R, HAM-D, HAM-A, BDI HAM-D, Pediatric TMS safety and tolerability measure	32 (13–21)	15	40 pulses over 4 seconds, 10 Hz; intertrain interval, 26 s; 75 trains; 3000 pulses	120	Headache, mild neck pain, unpleasant tingling, nausea, lightheadedness	56% responders responded and 44% achieved remission
Pan et al. [38]	Case series from a previous double-blind sham-controlled study	SCID BSI-CV MADRS	3 (16, 17, 15)	7	120 trains of 5 seconds dura­tion at 10 Hz with intertrain intervals of 15 seconds (i.e., 6000 pulses per session	100	Sleepiness, hypomanic symptoms	40-100% improvement in BSI-CV score, and 14.63–47.83% improvement in MADRS score of all three patients. Remission of suicidal thought of one of the patients
Wall et al. [28]	Open level study	KSADS-PL, CDRS-R, QIDS-A17-SR, CSSR-S, CGI-S, CGI-I	10 (13.9–17.4)	30	4-second stimulus trains, 10 HZ separated by 26-second intertrain intervals, with 3000 magnetic pulses per session	120	Scalp pain, worsening of depression, headaches, dizziness, musculoskeletal discomfort, neck stiffness, eye twitching, and nausea	60% of participants responded. The total mean score of CDRS-R, QIDS-A17-SR, and CGI-S significantly improved at 20, 30, and at 6-month follow-up
Croarkin et al. [29]	Open level	K-SADS, SCID, CDRS-R, ATHF	10 (13–17)	30 Six completed 30, and one each completed 1, 5, 17, and 29 sessions	4-second stimulus trains, 10 Hz separated by 26-seconds intertrain intervals, with 3000 magnetic pulses per session	120	Unspecified	~33.5% improvement in CDRS-R score post-treatment, and almost 45.6% improvement at 6 months post-treatment
Yang et al. [39]	Case series	KSADS, HAMD, BDI, HAMA	6 (15–21)	15	4 seconds stimulus, intertrain interval 26 seconds, 10 Hz, 3000 pulses per treatment	120%	Mild scalp discomfort, headache	66% responded 68% decrease in HAMD score and 84% reduction in BDI score 78% reduction of HAMA score
Wall et al. [30]	Open level, prospective, multi-center	KSADS-PL, CDRS-R, ATHF, CAVLT-2, D-KEFS, trail-making test	18 (13.9–17.8) 14 completed treatment	30	4 seconds stimulus duration, intertrain interval of 26 seconds, 10 Hz, 3000 stimuli per session	120%		Substantial improvement in depressive symptoms
Wall et al. [31]	Open level, perspective	K-SADS-PL, CDRS-R, QIDS-A17, CGI-S and CGI-I, CSSR-S, Subjective Reactive Questionnaire, Adverse Event Monitoring form	8 (14.6–17.8)	30	4 seconds stimulus duration, 10 Hz, intertrain interval 26 seconds, 75 trains, 3000 stimulations per session	120	Scalp pain	Almost 50% decrease in the mean baseline CDRS and QIDS A17 from baseline to at treatment 30
Croarkin et al. [40]	Case series	K-SADS-PL, CDRS- R	2 cases* (10 and 17)	Unspecified	Unspecified	Up to 40	Scalp pain	Unspecified
Bloch et al. [32]	Open level study	SCID, BDI, CDRS, CGI-S, SIQ, SCARED, CNTA	9 (16–18)	14	2-second trains, 10 Hz, intertrain interval 58 seconds, 20 stimuli, 400 stimuli per session	80%	Headache	Subjects had lower depression, anxiety, and no cognitive changes or suicidal ideation
Loo et al. [41]	First two cases from double-blind sham-controlled trial	MDRAS, BDI, CGI-S, CES- DC, RAVLT, Weschler digit span forward and backward, TMT A and B, COWAT	2 (16)	Case 1 - 29 Case 2 - 27	5 seconds, 10 Hz, 25 seconds intertrain interval, 40 trains, 2000 stimuli per session	110%	None	No cognitive changes
Walter et al. [44]	Emailed questionnaire to a worldwide pool of rTMS centers	HAMD, BQ	3 (16, 17, 17)	10	One case with - 2 seconds stimulus duration, 10 Hz, intertrain interval 28 seconds, 40 trains, 1600 stimulations per session. Two other cases - 8 seconds stimulus duration, 10 Hz, intertrain interval 52 seconds, 20 trains, 1600 stimulus per session			

**Table 2:** Study design, intervention, and efficacy case reports RDLPFC: right dorsolateral prefrontal cortex, LDLPFC: left dorsolateral prefrontal cortex, PPS: pulses per session, SCID: severe combined immunodeficiency.

Author	Rating scales	Age (years)	Number of sessions	Session duration (second per train, second per duration)	Intensity %MT	Reported side effects	Efficacy/effectiveness
Cullen et al. [33]	None	17	8	2-second trains, 18 Hz, 55, total 1980 pulses	85–120	Scalp pain Generalized, tonic-clonic seizure	Unspecified
Cristancho et al. [34]	No rating scale used	15	10 at RDLPFC 26 at LDLPFC	1 Hz, 10 seconds on, and 10–15 seconds off. The pulse increased from 150 PPS and increased to 300 PPS by the second week over RDLPFC and then 300–600 PPS by the fourth week over LDLPFC	90	Mild headaches, jaw twitch, and transient dizziness	Improvement in mood, less tearfulness
Segev et al. [35]	BDI-II, CDRS-R	17	20	4-second stimulus, intertrain interval 30 seconds, 10 Hz, 1680 pulses per treatment	100	Headache, scalp pain, and scalp burning	Improvement in anxiety from 46 to 25 score in SCARED. No improvement in BDI-II and CDRS-R scores. SIQ score improved transiently.
Chiramberro et al. [36]	SCID	16	12	5-second stimulus duration, 10 Hz, 60 trains, intertrain interval - 25 seconds, 3000 stimuli per session	Unspecified	Seizure	Unspecified
Hu et al. [37]		15	1	4-second stimulus duration, 10 Hz, intertrain interval 26 seconds, 20 trains per session, the cumulative number of daily pulses 800	80	The generalized tonic-clonic seizure followed by hypomanic symptoms lasting for 8-9 hours	Unspecified

The sample size ranged from 1 (case reports) to 103 [43]. The case series by Croarkin et al. presented three cases [40]. However, one of the three cases was a healthy control. So, we have included two out of these three cases in this systematic review. Similarly, the case series by Walter et al. presented seven cases. However, we included three of them as the other four subjects had other primary diagnoses such as bipolar disorder and schizophrenia [44]. All the multi-subject trials allowed comorbid anxiety disorder, dysthymia, attention deficit hyperactivity disorder (ADHD) but excluded schizophrenia, bipolar disorder, substance use disorder, post-traumatic stress disorder (PTSD), intellectual disability, pervasive developmental disorders, and eating disorders. One study included comorbid substance abuse [32], and another one allowed the inclusion of bipolar type II [42]. Zhang et al. included “mood or anxiety disorder” and thus, incorporated other diagnoses such as bipolar type II, dysthymia, generalized anxiety disorder [42]. No change in psychotherapy was allowed a few weeks before and during the trial [30-32]. Some studies have excluded patients with suicide attempts in the previous six months [28,31] or with “suicidal propensity” [42].

Most of the studies included treatment-resistant depression [25-32,39,43] and established the diagnosis by Kiddie Schedule for Affective Disorder and Schizophrenia [27-31,39,40]. Others utilized Structured Clinical Interviews based on the Diagnostic and Statistical Manual of Mental Disorders, fourth edition (DSM-IV) [32,36,38]; clinical interviews [35,42,43], Mini International Neuropsychiatric Interview for children, or Mini Neuropsychiatric Interview [43]. One of the studies used the World Health Organization International Classification of Disease - version 10 [37].

The outcomes were measured using Hamilton Depression Rating Scale (HAM-D) and Hamilton Anxiety Rating Scale (HAM-A) [27,39,42], HAM-D [43], Montgomery-Asberg Depression Rating Scale [38,41,43], Children’s Depression Rating Scale, Revised [28,29,31,32,35,43], Quick Inventory for Depressive Symptomatology Adolescent Seventeen Item Self Report [28,31,43], Clinical Global Impressions-Severity [28,31,32,41,43], Screen for Child Anxiety-Related Disorders questionnaire [32,35], Beck Depression Inventory [32,35,39,41], Beck Scale for Suicide Ideation-Chinese Version [38], Columbia Suicide Severity Rating Scale [28,31,43], Suicidal Ideation Questionnaire [32,35], Mini International Neuropsychiatric Interview [43], Mini International Neuropsychiatric Interview for Children [43], and Young Mania Rating Scale [43].

Antidepressant Use Among the Included Studies

The antidepressant use of the included studies is summarized in Tables 3 and 4. All the studies, except Croarkin et al., allowed antidepressant medications [43]. The studies have included patients with a history of one [27-30,39], two [32] to several failed antidepressant trials [28,33,40,43]. Pan et al. included either drug naïve or patients not taking antidepressants for nearly two weeks [38]. The majority of the studies have specified no change in antidepressant dosages during the trial [28-32,42]. Participants of some studies did not take any antipsychotics, mood stabilizers, benzodiazepines, stimulants, tricyclic antidepressants, or bupropion during TMS treatment [28,31].

**Table 3:** Antidepressant use and psychosocial intervention during the trial multi-subject trials SSRI: selective serotonin reuptake inhibitor; SNRI: serotonin and norepinephrine reuptake inhibitor, ATHF: Antidepressant Treatment History Form, TCA: tricyclic antidepressants.

Author	Antidepressant use	Previous treatment failure	Psychosocial intervention used	Change of dosages allowed
Croarkin et al. [43]	None	Yes	No change in psychosocial interventions	N/A
Zhang et al. [42]	All used one type of antidepressant treatment during rTMS along with other medications	Unspecified but excluded patients who were taking more than one antidepressant	Unspecified	No
MacMaster et al. [27]	Psychotropic medications use were allowed if stable for 6 weeks 31.25% were not on psychotropics	At least two previous failed medication trials	Unspecified	No unless medically necessary
Pan et al. [38]	Either drug naïve or was not taking antidepressants for 2 weeks prior to the study	All three patients took an antidepressant in the past for an unspecified dose and duration	Unspecified	N/A
Wall et al. [28]	All used either SSRI or SNRI	At least one prior medication trial failure as defined by ATHF. Study participants had a mean of 4.0 prior failed medication trial (SD 2.1).	No change in psychotherapy treatment was allowed within the last 4 weeks of treatment	No
Croarkin et al. [29]	All were on antidepressants	At least one prior failed medication trials	Unspecified	No
Yang et al. [39]	Unspecified	At least one failed trial	Unspecified	Unspecified
Wall et al. [30]	All were on antidepressants	At least one prior failed trial	No change in psychotherapy or therapist	No
Wall et al. [31]	Yes	Yes - at least two failed trials	No change in psychotherapy or therapist in 4 weeks prior to rTMS	No
Croarkin et al. [40]	Case 1 - none; Case 2 - yes	Case 1 - none; case 2 - multiple	Unspecified	No
Bloch et al. [32]	Yes	Failure of one course of psychotherapy and two courses of medication trials 8 weeks each, with at least one of the antidepressants being Fluoxetine	No change in therapy within 5 weeks before starting of rTMS	No
Loo et al. [41]	Case 1 - unspecified; case 2 - yes	Case 1 - failure of one course of psychotherapy; case 2 - one course of psychotherapy, two medication trials	Unspecified	Case 1 - unspecified; Case 2 - no
Walter et al. [44]	No	Unspecified	Unspecified	Unspecified

**Table 4:** Antidepressant use and psychosocial intervention during the trial case reports

Author	Antidepressant use	Previous treatment failure	Psychosocial intervention used	Change of dosages allowed
Cullen et al. [33]	None	Multiple failed trials	Unspecified	N/A
Cristancho et al. [34]	Yes	Multiple failed trials	Unspecified	N/A
Segev et al. [35]	Yes	Yes	Unspecified	Unspecified
Chiramberro et al. [36]	Yes	Yes	Unspecified	Unspecified
Hu et al. [37]	Yes on sertraline	Unspecified	Unspecified	Unspecified

Administration of rTMS

All studies delivered rTMS over the left dorsolateral prefrontal cortex (LDLPFC), except one study by Cristancho et al. that delivered it in both right and left DLPFC [34]. Most of them utilized the “5 cm rule” for localization of LDLPFC. Most of the studies used a figure of eight coil [27,34-39,41,42], and others used circular coil [32] or H1 coil [33]. The number of sessions varied from 1 [37,40] to 30 [28-31,43]. Most of the studies delivered rTMS at a frequency of 10 Hz, except two studies, Cullen et al. (18 Hz) and Cristancho et al. (1 Hz) [33,34]. Seven studies delivered 3000 stimuli per session [27-31,36,39,43]. The majority of the studies used 120% of motor threshold (MT) [27-31,39,42,43] and others varied from 40% to 120%, such as 100% [35,38], 90% [34], 80% [37], 80% [32], 110% [41], and 90-110% [44].

Efficacy/Effectiveness

The majority of the studies found that rTMS is a safe and effective add-on treatment for child and adolescent depression [27-32,34,38,39,42]. The improvement started as early as one [32,38] to two weeks [28,42]. Treatment benefits were noted at six months [28-31] to one year [32] post-trials. Croarkin et al. reported an improvement of 33.5% in CDRS-R scores and almost 45.6% improvement at six months post-treatment [29]. A similar rate of improvement (33%) was reported by Bloch et al. [32]. Wall et al. noted improvement among 60% of the study subjects [28]. Zhang et al. compared 42 adolescents with 75 adults and found a response rate of 94.1% and a remission rate of 88.2% among adolescents [42]. The older group had a less impressive improvement, higher baseline depression and anxiety scores, and shorter follow-up. A secondary analysis by Zhang et al. indicated that add-on rTMS improved depression and somatic and psychic anxiety among adolescents [24]. Sonmez et al. pooled data from three previous studies [28,30,31] and found that rTMS may improve hypersomnia in addition to its antidepressant action [26].

Side Effects

The majority of the studies reported mild side-effects such as scalp tenderness and headache [27,28,31-35,39,42-44], musculoskeletal discomfort [28,42], neck-pain and stiffness [27,28]. The other side effects that were reported include eye-twitching [28], eye pain [43], facial twitch [43], jaw-twitching [34], nausea [27,28,43], tingling sensation [27], and lightheadedness/dizziness [27,28,34]. There are three case reports of treatment-emergent seizures [33,36,37] and one case series of intolerable headaches [40] leading to the termination of the procedure. There is one case report of emergent hypomanic symptoms [38]. No cognitive decline was reported [30,31].

Suicidal Ideation

Several studies have assessed suicidal behavior and found no worsening of symptoms during rTMS. Croarkin et al. reported that 4 out of 103 patients developed suicidal ideation, but none were related to rTMS treatment [43]. Wall et al. reported 8 out of 10 subjects had suicidal ideation before the study, two subjects experienced worsening suicidal thoughts during the trial [28]. One subject had self-injurious behavior at six months follow up. In another study, subjects with suicidal thoughts at baseline reported improvement with treatment [31]. At the end of six months, one patient had self-mutilating behavior, and another patient needed hospitalization, which was unrelated to rTMS. In a pooled data analysis of the above studies [28,31], the suicidal ideation at baseline and at the end of the study period was 63.16% and 16.67%, respectively [25]. In another case series by Pan et al., one out of three subjects experienced remission of suicidal ideation during rTMS [38]. Segev et al. reported a case where suicidality was transiently improved, followed by deterioration, and finally plateaued [35]. Bloch et al. also found out that suicidal ideation was not affected by the stage of rTMS treatment [32].

Discussion

To the best of our knowledge, our systematic review is the only one that has incorporated the data of more than 250 patients. Our findings support the results of the previous systematic reviews on the topic [45,46]. Previous systematic reviews on this topic have been criticized for lacking a controlled group in the included studies and the relatively smaller number of total subjects in the included studies [45,46]. In our systematic review, the addition of the only available RCT with a sham-control group improved the quality of the evidence of efficacy by utilizing a comparison group that approximates a placebo group [47]. Our systematic review included the three recent studies with larger sample sizes than previous articles [27,42,43]. Although better quality studies are still needed, there is a strong indication that rTMS is a safe and useful add-on, but not a stand-alone treatment for child and adolescent depression that has not responded to at least one antidepressant treatment. Twelve out of 18 included studies found out that rTMS could be an effective treatment for major depression among children and adolescents.

Studies showing reduced depressive symptoms have most commonly delivered rTMS in 10 Hz frequency, 120% MT, four seconds train, and 3000 stimuli per session for 30 sessions. One of the significant advantages of rTMS is that the patient can be monitored multiple times a week. Moreover, the side- effects are mild and self-limiting. Suicidality and treatment-emergent manic or hypomanic symptoms appear to be rare with rTMS in this age group. The side effects reported in the majority of the studies are mild and self-limiting. Out of the three case reports demonstrating the development of seizure spells in the context of rTMS, one delivered rTMS in a somewhat different protocol from the other studies, such as they used two-second trains (instead of the usual four-second trains used by other authors), used 18 Hz frequency instead of 10 Hz frequency, and delivered a total of 1980 pulses per session [33]. The other case report of seizure spell involved the use of 75 mg olanzapine (which was later corrected by the author as 7.5 mg) and a blood alcohol concentration of 0.20% at the time of the seizure episode [36]. The only one case report of the emergence of hypomanic symptoms has used 6000 pulses per session [38]. Such a low occurrence of significant side effects has also been reported in other non-invasive brain stimulation studies involving children and adolescents [48-50].

According to the University of Oxford Centre for Evidence-Based Medicine published criteria to determine the evidence levels, the sham-controlled RCT demonstrated level 1 evidence that rTMS is safe but is not better than placebo as a stand-alone treatment for resistant depression among children and adolescents [23]. Nevertheless, 41.7% responded, and 29.2% achieved remission with rTMS [43]. Seven open-label studies showed level 2 evidence for efficacy [27-32,42]. Multiple case series and case reports showed a level 4 degree of evidence of efficacy [34-41,44]. The study's treatment response rate by Zhang et al. seems to be an outlier. It could be because of the naturalistic study design or having a somewhat different inclusion and exclusion criteria [42]. Further, the study's comparison groups were different from each other in significant parameters such as the course of illness, baseline HAM-D score [42]. The studies that did not find rTMS as a useful tool for the treatment of depression have either used it as a stand-alone treatment [43] or comprise of only case reports demonstrating side-effects that led to premature termination of treatment [36,37,40].

We have used Joanna Briggs Institute Critical Appraisal Tools to assess the quality of the included studies (see Appendix) [22]. The overall quality of the studies is average except for Croarkin et al. which seems to have high [43]. rTMS can still be considered a relatively novel treatment option for minors and could have contributed to the included studies’ relatively small sample sizes. Moreover, almost all the studies have used well-validated diagnostic tools and rating scales to provide objective data of the treatment response. All the studies attempted to use rTMS only in the context of previous treatment failures. Still, the existing database's main drawbacks are the studies' limited sample size and mostly open-label study designs. The overall quality of the existing studies is low. The other limitations are heterogeneity in the inclusion criteria, the number of previously failed antidepressant trials, various parameters, and the number of rTMS sessions. The heterogeneity mentioned above could explain some varied treatment responses in the reported studies. There is a strong need to conduct RCTs involving large study subjects to explore the efficacy of rTMS as an add-on treatment in child and adolescent major depression. There is a need to explore the stage of treatment of Major Depression when the addition of rTMS to antidepressant treatment could lead to the best possible treatment outcome in terms of its utility as an add-on agent for treatment. This is especially true given the favorable side effects reported in the previously published studies. Although it is challenging to draw a sweeping conclusion at this time, rTMS has a strong potential in treating depression among children and adolescents, particularly among adolescents.

## Conclusions

Major depression among children and adolescents is associated with a high non-response rate with traditional treatment. rTMS can be a useful add-on treatment for child and adolescent major depression, not responding to at least one antidepressant. The existing database is limited by the limited number of RCTs. Despite limitations, most of the studies indicate that rTMS is generally safe among children and adolescents and carries a minimal risk of seizure and treatment-emergent hypomanic symptoms. rTMS is not more effective than placebo as a stand-alone treatment of resistant major depression among children and adolescents.

## Appendices

**Table 5:** Joanna Briggs institute quality appraisal tool for case reports Yes= +, No= -, Unclear= ?, Not applicable= NA. Q1. Were the patient’s demographic characteristics clearly described? Q2. Was the patient’s history clearly described and presented as a timeline? Q3. Was the current clinical condition of the patient on presentation clearly described? Q4. Were diagnostic tests or assessment methods and the results clearly described? Q5. Was the intervention(s) or treatment procedure(s) clearly described? Q6. Was the post-intervention clinical condition clearly described? Q7. Were adverse events (harms) or unanticipated events identified and described? Q8. Does the case report provide takeaway lessons?

	Q1	Q2	Q3	Q4	Q5	Q6	Q7	Q8
Cullen et al. [33]	-	-	-	+	-	-	+	+
Cristancho et al. [34]	+	+	+	-	+	-	+	+
Segev et al. [35]	+	+	+	+	+	+	+	+
Chiramberro et al. [36]	-	+	+	+	+	-	+	-
Hu et al. [37]	-	-	-	-	+	+	+	+

**Table 6:** Joanna Briggs institute quality appraisal tool for quasi-experimental studies (non-randomized experimental studies) Yes= +, No= -, Unclear= ?, Not applicable = NA. Q1. Is it clear in the study what is the ‘cause’ and what is the effect’ (i.e., there is no confusion about which variable comes first)? Q2. Were the participants included in any comparisons similar? Q3. Were the participants included in any comparisons receiving similar treatment/care, other than the exposure or intervention of interest? Q4. Was there a control group? Q5. Were there multiple measurements of the outcome both pre and post the intervention/exposure? Q6. Was follow-up complete and if not, were differences between groups in terms of their follow-up adequately described and analyzed? Q7. Were the outcomes of participants included in any comparisons measured in the same way? Q8. Were outcomes measured in a reliable way? Q9. Was appropriate statistical analysis used?

	Q1	Q2	Q3	Q4	Q5	Q6	Q7	Q8	Q9
Zhang et al. [42]	+	-	+	-	+	+	+	+	+
MacMaster et al. [27]	+	+	+	-	+	+	+	+	+
Wall et al. [28]	+	+	+	-	+	+	+	+	+
Croarkin et al. [29]	+	+	+	-	+	+	+	+	+
Wall et al. [30]	+	+	+	-	+	+	+	+	+
Wall et al. [31]	+	+	+	-	+	+	+	+	+
Bloch, 2008 [32]	+	+	+	-	+	+	+	+	+

**Table 7:** Joanna Briggs institute quality appraisal tool for randomized controlled trials Yes= +, No= -, Unclear= ?, Not applicable = NA. Q1. Was true randomization used for the assignment of participants to treatment groups? Q2. Was allocation to treatment groups concealed? Q3. Were treatment groups similar at the baseline? Q4. Were participants blind to treatment assignment? Q5. Were those delivering treatment blind to treatment assignment? Q6. Were outcomes assessors blind to treatment assignment? Q7. Were treatment groups treated identically other than the intervention of interest? Q8. Was follow-up complete and if not, were differences between groups in terms of their follow-up adequately described and analyzed? Q9. Were participants analyzed in the groups to which they were randomized? Q10. Were outcomes measured in the same way for treatment groups? Q11. Were outcomes measured in a reliable way? Q12. Was appropriate statistical analysis used? Q13. Was the trial design appropriate, and any deviations from the standard RCT design (individual randomization, parallel groups) accounted for in the conduct and analysis of the trial?

	Q1	Q2	Q3	Q4	Q5	Q6	Q7	Q8	Q9	Q10	Q11	Q12	Q13
Croarkin et al. [43]	+	+	+	+	+	+	+	+	?	+	+	+	+

**Table 8:** Joanna Briggs institute quality appraisal tool for case series Yes= +, No= -, Unclear= ?, Not applicable= NA. Q1. Were there clear criteria for inclusion in the case series? Q2. Was the condition measured in a standard, reliable way for all participants included in the case series? Q3. Were valid methods used for the identification of the condition for all participants included in the case series? Q4. Did the case series have consecutive inclusion of participants? Q5. Did the case series have complete inclusion of participants? Q6. Was there clear reporting of the demographics of the participants in the study? Q7. Was there clear reporting of clinical information of the participants? Q8. Were the outcomes of follow-up results of cases clearly reported? Q9. Was there clear reporting of the presenting site(s)/clinic(s) demographic information? Q10. Was statistical analysis appropriate?

	Q1	Q2	Q3	Q4	Q5	Q6	Q7	Q8	Q9	Q10
Pan et al. [38]	+	+	+	-	-	-	+	+	-	NA
Yang et al. [39]	+	+	+	?	+	-	-	+	-	+
Croarkin et al. [40]	-	+	+	-	-	-	+	+	-	NA
Loo et al. [41]	+	+	+	+	-	-	+	+	-	?
Walter et al. [44]	-	+	+	-	-	-	-	+	-	-
